# Gouty tophi in Domenico Fetti’s *Archimedes*: a clinico-iconographic analysis

**DOI:** 10.1007/s40618-026-02867-7

**Published:** 2026-03-28

**Authors:** Amelian Madalin Bobu, Andrei Ionut Cucu, Raffaella Bianucci

**Affiliations:** 1 Faculty of Medicine, “Grigore T. Popa” University of Medicine and Pharmacy, Iași, Romania; 2 ISI, International Society of Iconodiagnosis, Paris, France; 3 Faculty of Medicine and Biological Sciences, University “Stefan cel Mare of Suceava”, Suceava, Romania; 4 Laboratory Anthropology, Archaeology, Biology (LAAB), Paris-Saclay University, Montigny- Le-Bretonneux, Yvelines, France

**Keywords:** Gout, Iconodiagnosis, Domenico Fetti, Archimedes

## Abstract

**Purpose:**

To propose a clinico-iconographic diagnosis of gouty tophi in Domenico Fetti’s painting *Portrait of a Scholar* (*Archimedes*) and to evaluate the morphological features in relation to differentialdiagnoses such as osteoarthritis and rheumatoid arthritis.

**Methods:**

A detailed visual and morphological analysis of the painting was conducted, focusing on the anatomical features of the depicted hand. The observed characteristics were interpreted within aclinical framework and compared with known manifestations of gout, osteoarthritis, and rheumatoid arthritis. The evaluation followed the International Guidelines for Iconodiagnosis.

**Results:**

The painting shows nodular formations at the level of the thumb, as well as the proximal and distal interphalangeal joints of the index finger and the interphalangeal joint of the third finger.These features are consistent with tophaceous deposits observed in chronic gout. The distribution and morphology of the nodules are less compatible with osteoarthritis and rheumatoid arthritis, basedon their typical clinical patterns.

**Conclusions:**

The clinico-iconographic correlation supports a retrospective diagnosis of probable chronic gouty arthritis, with a Level of Evidence II. This study highlights the value of artworks as potentialhistorical sources for medical observation, bridging art history and clinical interpretation.

Gout, whose name derives from the Latin *gutta* (“drop”), reflects the medieval belief that an excess of one of the four humors would “drip” into the joints, causing pain and inflammation. Today, it is defined as a disorder of uric acid metabolism characterized by hyperuricemia and recurrent episodes of acute inflammatory arthritis. Chronic untreated hyperuricemia may lead to the deposition of monosodium urate crystals in soft tissues, forming tophaceous deposits that can become clinically visible as nodular lesions around joints [1]. Historically associated with rich diets and excessive alcohol consumption, it was known as “the disease of kings,” reflecting its perceived link to privileged lifestyles [2].

Within this nosological and historical framework, we propose a possible iconographic representation of gouty arthritis in the painting by Domenico Fetti (c. 1589–1623) entitled „Portrait of a Scholar (Archimedes)” and reproduced in Fig. 1a. Active mainly in Mantua at the Gonzaga court, Fetti developed a style influenced by Flemish painters (Rubensian style) characterized by dramatic chiaroscuro and refined realism. His artworks demonstrate a marked interest in physiognomic detail, as seen in *The Young Beggar*, where morphological precision has been interpreted as suggestive of underlying endocrine pathology [3].

**Fig. 1:**
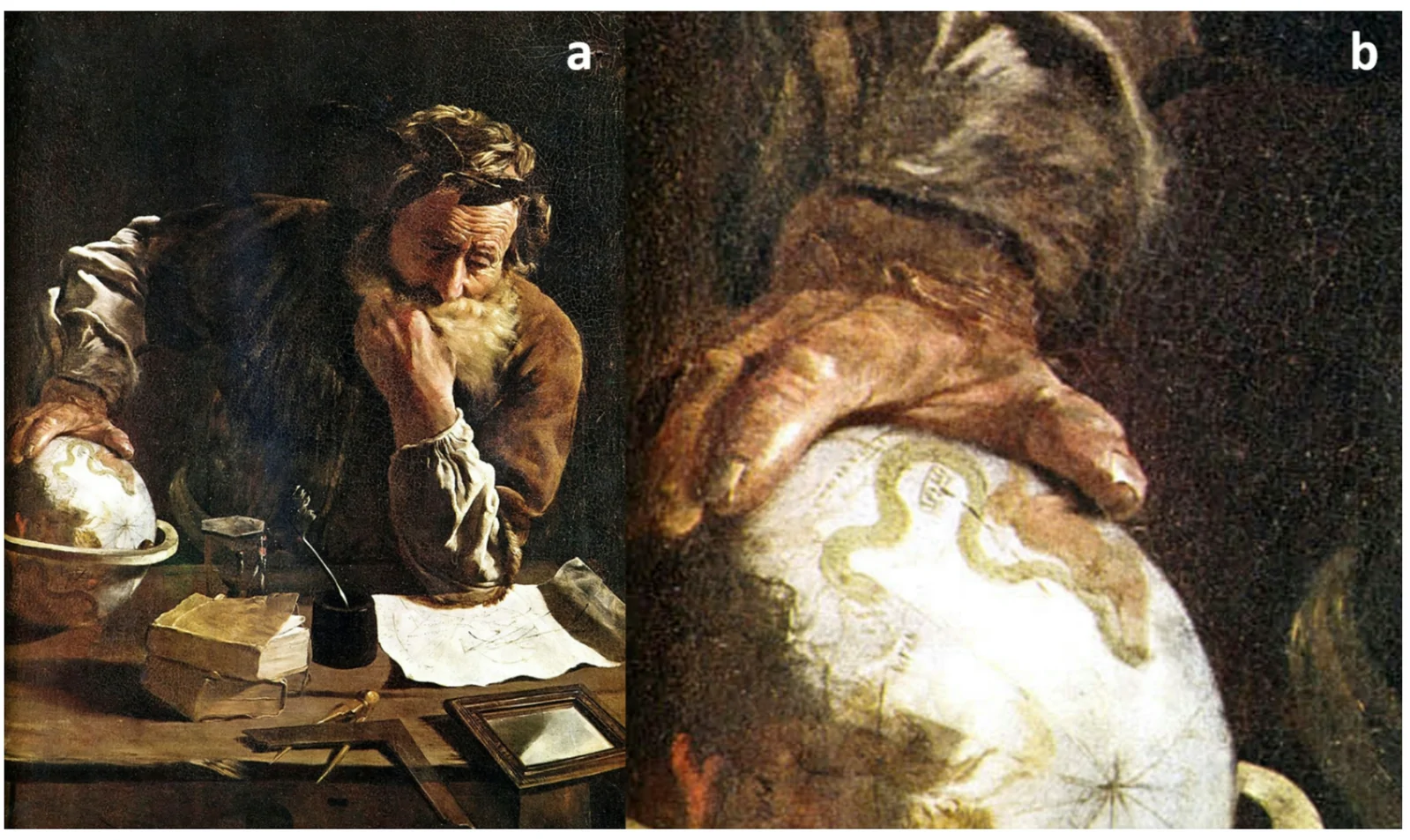
Domenico Fetti, Portrait of a Scholar (Archimedes), oil on canvas, circa 1620, height 98 cm (38.5 in), width 73.5 cm (28.9 in), Staatliche Kunstsammlungen Dresden, Germany; **b** detail showing gouty tophi level of the thumb, at the proximal and distal interphalangeal joints of the index finger, and at the interphalangeal joint of the third finger (public domain)

Painted during Fetti’s Mantuan period, *Archimedes*, likely remained in Italy until 1743, when it entered the collection of Augustus III of Saxony. Later interventions, including the addition of an approximately 10 cm strip at the lower edge, indicate posthumous modifications; a possible contribution by Guercino (Giovanni Francesco Barbieri, 1591–1666) has also been hypothesized (Fig. 1a).

From a compositional perspective, *Archimedes* is portrayed as an elderly man leaning over his desk, absorbed in mathematical formulas. The terrestrial globe on which he rests his right hand symbolizes the universality of knowledge; however, it is precisely this hand that becomes central to the medical analysis. A close examination of the pictorial details reveals nodular formations suggestive of gouty tophi, apparently located at the level of the thumb, the proximal and distal interphalangeal joints of the index finger, and the interphalangeal joint of the third finger (Fig. 1b). The appearance of these protrusions, rendered with meticulous care, suggests a realistic observation, possibly inspired by a live model.

The principal differential diagnoses include osteoarthritis (OA) and rheumatoid arthritis (RA). Digital OA presents with Heberden’s and Bouchard’s nodes at the interphalangeal joints, typically without significant inflammatory signs. RA more commonly involves the metacarpophalangeal and proximal interphalangeal joints in a symmetrical distribution, often accompanied by characteristic deformities, features not evident in this representation. In contrast, chronic gout may produce visible tophaceous deposits consistent with the nodular morphology depicted.

Based on morphological assessment and clinico-iconographic correlation, we propose a retrospective diagnosis of probable chronic gouty arthritis. Applying the International Guidelines for Iconodiagnosis, we performed a systematic evaluation of the visible features and assigned a Level of Evidence II, reflecting detailed iconographic analysis supported by solid clinical reasoning but lacking direct documentary confirmation [4]. This case underscores the potential of artwork as a historical document of disease, situated at the intersection of medical observation and aesthetic expression.
